# “Arsenic in Food”: Silbergeld Responds

**Published:** 2005-04

**Authors:** Ellen Silbergeld

**Affiliations:** Johns Hopkins University, Bloomberg School of Public Health, Baltimore, Maryland, E-mail: esilberg@jhsph.edu

Bernard comments on the calculations in my letter (Silbergeld 2004) to criticize my conclusions, which were that the use of arsenic for growth promotion in poultry feeds results in contamination of chicken products (and other food animal products because of the use of poultry litter in feeds), and that the estimates of risk have not been adequately calculated, even by Lasky et al. (2004) because of unsubstantiated inferences of the arsenic concentrations in edible tissues and a puzzling use of outdated risk assessments for arsenic. I find it interesting that Bernard (who has consulted for the Food and Drug Administration, the agency that permits this nontherapeutic use of arsenicals in animal feeds) does not comment on these conclusions in his letter.

I acknowledge the error in quoting Lasky et al. (2004); I used the wrong metric in quoting her conclusions. Please do not ascribe responsibility to my colleagues, who read this letter in manuscript form, or to *EHP*’s editorial office. However, I do not agree that this mistake invalidates the conclusions of my letter. If the concentrations of arsenic in edible chicken meat are not one-tenth of those in liver (as claimed by Alpharma, the manufacturer of roxarsone), then the exposure of Americans who consume chicken (such as my son, who appeared to exist largely on chicken wings during high school) is in fact 3–10 times higher than Lasky et al. estimated, resulting in an intake of 4–50 μg/day. This is still in excess of the current National Research Council (NRC) recommendation (NRC 2001).

This risk estimate does not include the potential for additional exposures to arsenic from confined animal feeding operation (CAFO) wastes via land disposal, which may reach human populations though soil contact, groundwater contamination, and plant uptake, as noted in my letter (Silbergeld 2004). These exposures may be important for regions such as the Eastern Shore, where between 600 and 800 million broiler chickens are raised each year. The U.S. Geological Survey has estimated that thousands of kilograms of arsenic may be land disposed with poultry wastes (Garbarino et al. 2003).

Given the article by Lasky et al. (2004) and new information on the environmental pathways of arsenic releases from CAFOs (Han et al. 2004; Jackson et al. 2003), as well as new studies on the health effects of arsenicals (Simeonova and Luster 2004), I suggest that it is time for a thoughtful consideration of the use of arsenicals as growth promoters in animal feeds.
